# A multi-institutional study of association of sonographic characteristics with cervical lymph node metastasis in unifocal papillary thyroid carcinoma

**DOI:** 10.3389/fendo.2022.965241

**Published:** 2022-09-23

**Authors:** Liuhua Zhou, Jincao Yao, Di Ou, Mingkui Li, Zhikai Lei, Liping Wang, Dong Xu

**Affiliations:** ^1^ Zhejiang Chinese Medical University, Hangzhou, China; ^2^ Cancer Hospital of the University of Chinese Academy of Sciences (Zhejiang Cancer Hospital), Hangzhou, China; ^3^ Institue of Basic Medicine and Cancer (IBMC), Chinese Academy of Sciences, Hangzhou, China; ^4^ Zhejiang Xiaoshan Hospital, Hangzhou, China; ^5^ Hangzhou Cancer Hospital, Hangzhou, China; ^6^ Key Laboratory of Head & Neck Cancer Translational Research of Zhejiang Province, Hangzhou, China; ^7^ Zhejiang Provincial Research Center for Cancer Intelligent Diagnosis and Molecular Technology, Hangzhou, China

**Keywords:** ultrasonography, thyroid cancer, papillary, lymphatic metastases, imaging, TI-RADS category

## Abstract

**Objective:**

Papillary thyroid carcinoma (PTC) is the most common pathological type of thyroid carcinoma, and is prone to cervical lymph node metastases (CLNM). We aim to evaluate the association between sonographic characteristics of PTC and CLNM before the initial surgery.

**Methods:**

Clinical information as well as ultrasonographic measurements and characteristics for 2376 patients from three hospitals were acquired in this retrospective cohort study. Univariate and multivariate logistic analysis were performed to predict CLNM in unifocal PTC patients. Receiver operating characteristic (ROC) curve was created to evaluate diagnostic performance.

**Results:**

Univariate analysis showed that gender, age, maximum tumor diameter and volume, cross-sectional and longitudinal aspect ratio, location, echogenicity, margin, and echogenic foci were independently associated with CLNM metastatic status (P < 0.05). Multivariate logistic analysis showed that gender, age, maximum tumor diameter and volume, cross-sectional aspect ratio (CSAR), location, echogenicity, margin, and echogenic foci were independent correlative factors; CSAR showed a significant difference for PTC2 to predict CLNM. The area under the curve (AUC) of the maximum tumor diameter, tumor volume, margin, and echogenic foci was 0.70, 0.69, 0.65, and 0.70, respectively. The multiple-variable linear regression model was constructed with an AUC of 0.77, a specificity of 73.4%, and a sensitivity of 72.3%. Kruskal-Wallis analysis for positive subgroups, maximum tumor diameter and volume, cross-sectional and longitudinal aspect ratio, margin, and echogenic foci showed statistical significance (P < 0.05).

**Conclusions:**

Younger age (< 55 years), male, larger tumor, and echogenic foci were high risk factors for CLNM in patients with unifocal PTC. CSAR had a more effective predictive value for CLNM in patients with larger thyroid tumors. A larger tumor with irregular and punctate echogenic foci was also more prone to the lateral neck, and both central and lateral neck metastasis.

## Background

As a noninvasive imaging examination method, ultrasonography is widely used in clinical settings. The thyroid imaging report and data system (TI-RADS) white paper, which was proposed by the American College of Radiology in 2017, has been internationally recognized and used recently (1). This paper presented the characteristics of thyroid nodules based on sonographic characteristics such as composition, echogenicity, shape, margin, and echogenic foci. TI-RADS regulates the classification diagnostic criteria, which provides better guidance in judgement of thyroid nodules.

Papillary thyroid carcinoma (PTC) is the most common pathological type of thyroid carcinoma, among which unifocal tumors often occur. The incidence of multifocal PTC is about 30% (2). CLNM is prone to occur early, and there is a risk of postoperative recurrence as well as distant metastasis (3). However, there are many limitations in ultrasonography of cervical lymph nodes, especially for CLNM, and ultrasonography has a detection rate of 18.8%–31.0%. This technique is limited because of interference from the trachea, esophagus, osseous tissue, and thyroid underlying diseases, as well as the experience of the examiner (4, 5). In recent years, preventive central lymph node dissection has been executed for PTC patients (6–8); however, according to the 2015 American Thyroid Association Management Guidelines (9), thyroidectomy without prophylactic central neck dissection is appropriate for small (T1 or T2), noninvasive, clinically node-negative PTC (cN0). We hope that high risk factors of PTC nodules with CLNM will be stratified in routine ultrasound examination, such that head and neck surgeons as well as radiologists can better assess and complete preoperative surgical planning. Then, prophylactic neck dissection (central/lateral) can be offered accordingly. This will be useful to guide treatment protocols prior to operating.

Ultrasonography is the primary inspection method for the thyroid gland, and nodular sonographic characteristics are easy to access through basic training. In this study, unifocal PTC was selected for analysis in combination with clinical information, ultrasonographic measurements, and characteristics. There is an urgent need to identify metrics for predicting the risk of lymph node metastasis before surgery when performing routine thyroid and cervical lymph node ultrasonic screening.

## Materials and methods

### Patients

The enrolled participants were symptomatic with palpable or incidental thyroid lumps, and all ultimately underwent thyroidectomy in Cancer Hospital of the University of Chinese Academy of Sciences (Zhejiang Cancer Hospital, Hangzhou, China), Zhejiang Xiaoshan Hospital and Hangzhou Cancer Hospital from July 2017 to September 2020. The inclusion criteria were as follows: 1) patients were undergoing first-time thyroidectomy; 2) patients received enhanced computed tomography (CT) scan of neck and thorax to assess cervical lymph node and pulmonary metastasis, as well as thyroid and neck ultrasonography before operation; and 3) PTC was confirmed by biopsy before surgery. The exclusion criteria were as follows: 1) identification of lung or other distant metastasis; and 2) postoperative pathology diagnosis of multifocal PTC. Unilateral thyroid lobe plus isthmus excision or total thyroidectomy were performed for each patient. If lateral lymph node metastasis was suspected by preoperative comprehensive evaluation and confirmed by biopsy, lateral lymph node dissection was performed (6). All patients underwent preventive central lymph node dissection (6–8) and metastatic lymph nodes were confirmed by pathology. This study was approved by the Ethics Committee of Cancer Hospital of the University of Chinese Academy of Sciences (Zhejiang Cancer Hospital), Zhejiang Xiaoshan Hospital and Hangzhou Cancer Hospital. Informed consent was obtained from all enrolled patients.

**Figure 1:**
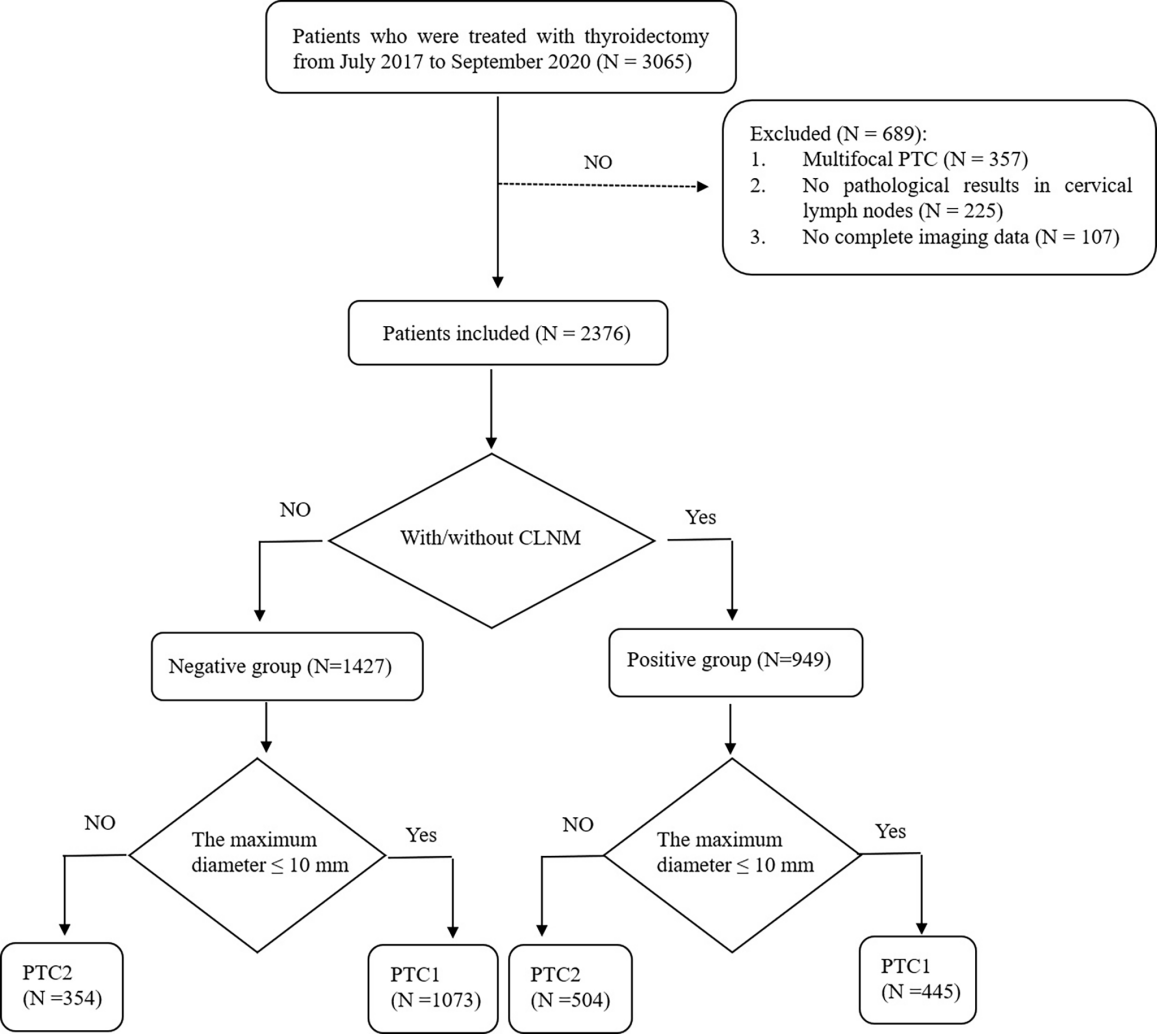
Flow diagram of the study selection procedure.

A total of 3065 patients undergoing thyroidectomy were enrolled. Of the 689 cases excluded from the study, 357 were multifocal PTC, 225 had no pathological results in cervical lymph nodes, and 107 had incomplete imaging data. Ultimately, we analyzed 2376 patients with unifocal PTC in the study. Patients were divided into the following two groups: (1) 949 patients with CLNM were placed in the positive group and (2) 1427 patients with no CLNM were placed in the negative group. For the positive group, subgroups of central lymph node metastasis, lateral lymph node metastasis, and both central and lateral lymph node metastasis were created. We also assigned papillary thyroid microcarcinoma (PTMC) as group PTC1 and PTC above pT1a as group PTC2 (**Figure 1**).

### Instruments

For examinations, we used the GE Logiq E9 ultrasonographic instrument (General Electric Healthcare, Milwaukee, WI, USA) with a high-resolution linear probe (ML6-15) and the Philips iU22 ultrasonographic instrument (Royal Dutch Philips Electronics, Amsterdam, Noord-Holland, Netherlands) with a high frequency linear probe (L12-5).

### Protocol

Patients were maintained in a supine position with the neck hyperextended while the thyroid and both sides of the neck were scanned in multi-section. The three diameters (length, width, and height), lesion location, composition, echogenicity, shape, margin, and echogenic foci of the tumor were recorded and evaluated from workstations. The patients were followed up until they underwent thyroidectomy and pathological results were obtained. The duration of follow-up ranged from 1 week to 2 months.

The ultrasonographic images were obtained by the same three professional physicians, each with more than 10 years of experience, board-certification, and training in thyroid ultrasound. The ultrasonographic images and reports were independently analyzed in a blinded fashion by another two ultrasound specialists (each with more than 10 years of experience). All imaging data were compared to the pathological results from neck dissections. For measurements, if the error was less than 2 mm between the two ultrasound specialists, the average value was applied. When the measurement error exceeded 2 mm, or if any other discordance occurred for ultrasonographic characteristics, a separate experienced sonographer (with more than 20 years of experience in thyroid ultrasound) reviewed the images and a final decision was reached.

Clinical information as well as ultrasonographic measurements and characteristics were used for data collection. Clinical information included gender and age. An age threshold of 55 years was used for analysis according to the 8th edition of the United States Joint Committee on Cancer, as the diagnostic age of the TNM staging system for thyroid cancer was 55 years (9). US measurements included the maximum tumor diameter, tumor volume, cross-sectional aspect ratio, and longitudinal aspect ratio.

The three diameters of the tumor were determined by: 1) completing a longitudinal scan of the thyroid, selecting the maximum section of the nodule, and measuring the maximum long diameter as the length; 2) measuring the vertical diameter of long diameter as the height; and 3) performing a transversal scan of the thyroid, selecting the maximum section of the nodule, and recording the maximum diameter from left to right as the width. The maximum diameter was the maximum of length, width and height, volume = 0.523 × length × width × height, cross-sectional aspect ratio = height/width and longitudinal aspect ratio = height/length.

Sonographic characteristics according to ACR TI-RADS were carried out as follows: composition (cystic, mixed cystic and solid, solid/almost completely solid); echogenicity (anechoic, hyperechoic or isoechoic, hypoechoic and very hypoechoic); shape (aspect ratio < 1 or ≥ 1); margin (smooth or ill-defined, lobulated or irregular, extra-thyroid extension); and echogenic foci (none or large comet-tail artifacts, macro, peripheral and punctate echogenic foci).

### Statistical analysis

The obtained data were statistically analyzed by IBM SPSS Statistics version 23 (IBM SPSS INC., Chicago, USA). Continuous quantitative data were expressed as the mean± standard deviation (SD). Data-counting were described statistically by the number of cases and rates. Chi-square test and independent-sample T test were used for univariate analysis. A multivariate analysis using binary logistic regression analysis was adopted if analysis index P < 0.05 in the univariate analysis.

Odds ratios (ORs) with 95% confidence intervals (CIs) were calculated and ROC curves were analyzed for factors with significance on binary linear regression analysis, AUC curves, sensitivity and specificity of each significant independent variable were acquired. Multiple linear regression equations reflect the relationship between independent variables and covariates; covariates are originated from binary linear regression analysis that conform to P < 0.05. Kruskal-Wallis analysis was used for the positive subgroups to find out the significance. P < 0.05 was considered statistically significant.

## Results

A total of 2376 patients with 2376 lesions were included (1518 PTC1 and 858 PTC2), and the clinical data and sonographic characteristics were retrospectively analyzed. There were 569 males and 1807 females, aged 12 to 84 years, with an average age of 44.5 ± 11.8 years; the maximum tumor diameter was 3.5 to 64.7 mm, with an average diameter of 10.4 ± 7.5 mm. A total of 949 cases were in the positive group (39.9% of total); 445 were PTC1 and 504 were PTC2. In the positive group, 669 (70.5%) patients had only central lymph node metastasis, 70 (7.4%) had only lateral lymph node metastasis, and 210 (22.1%) had metastasis in both central and lateral lymph nodes. There were 1427 cases included in negative group (60.1% of total); 1073 were PTC1 and 354 were PTC2. Of these, 227 patients underwent lateral lymph node dissection due to a positive preoperative biopsy. Lateral neck lymph node dissection was performed in seven cases with high imaging suspicion but negative puncture results. Four of these cases were found to have lateral neck lymph node metastasis and three were confirmed negative during operation.

For the positive and negative groups, the results showed that male (x2 = 37.914, P < 0.001), age (t = 13.342, P < 0.001), age < 55 years (x2 = 33.608, P < 0.001), maximum tumor diameter (t = -17.431, P < 0.001) and volume (t = -9.051, P < 0.001), cross-sectional aspect ratio (t = 6.926, P < 0.001), and longitudinal aspect ratio (t = 9.343, P < 0.001) were all significantly related to CLNM (**Table 1**).

**Table 1:** Univariate analysis of nodular parameters with CLNM in PTC.

Variable	Positive group (n=949)	Negative group (n=1427)	Statistics	P
Gender (male/female)	290/659	279/1148	x^2^ = 37.914	P < 0.001
Age	41.1 ± 12.0	46.6 ± 11.3	t=13.342	P < 0.001
Age (≥ 55 years/< 55 year)	139/810	349/1078	x^2^ = 33.608	P < 0.001
Tumor maximum diameter (mm)	13.3 ± 9.1	8.5 ± 5.6	t=-17.431	P < 0.001
Tumor volume (ml)	1.6 ± 4.0	0.5 ± 2.1	t=-9.051	P < 0.001
Cross-sectional aspect ratio	1.0 ± 0.3	1.1 ± 0.3	t=6.926	P < 0.001
Longitudinal aspect ratio	0.9 ± 0.3	1.0 ± 0.3	t=9.343	P < 0.001

By comparing sonographic characteristics of patients in the positive and negative groups using the Chi-square test and the independent-sample T test, our results showed that location, echogenicity, aspect ratio, margin, and echogenic foci showed significant differences (**Table 2**).

**Table 2:** Univariate analysis of sonographic features with CLNM in PTC.

Variable	Positive group (n=949)	Negative group (n=1427)	Statistics	P
**Location**			x^2^ = 34.052	P<0.001
Left upper	117 (12.3)	196 (13.7)		
Left middle	195 (20.6)	364 (25.5)		
Left lower	88 (9.3)	119 (8.4)		
Right upper	175 (18.4)	223 (15.6)		
Right middle	203 (21.4)	366 (25.7)		
Right lower	112 (11.8)	100 (7.0)		
Isthmus	59 (6.2)	59 (4.1)		
**Composition**			x^2^ = 0.281	P=0.596
Mixed cystic and solid	10 (1.1)	12 (0.8)		
Solid or almost completely solid	939 (98.9)	1415 (99.2)		
**Echogenicity**			x^2^ = 18.186	P<0.001
Hyperechoic or isoechoic	16 (1.7)	26 (1.8)		
Hypoechoic	856 (90.2)	1344 (94.2)		
Very hypoechoic	77 (8.1)	57 (4.0)		
**Aspect ratio**			x^2^ = 28.974	P<0.001
<1	451 (47.5)	520 (36.4)		
≥1	498 (52.3)	907 (63.6)		
**Margin**			x^2^ = 255.392	P<0.001
smooth or ill-defined	533 (56.2)	1221 (85.6)		
lobulated or irregular	359 (37.8)	182 (12.7)		
extra-thyroid extension	57 (6.0)	24 (1.7)		
**Echogenic foci**			x^2^ = 368.018	P<0.001
none or large comet-tail artifacts	328 (34.6)	1049 (73.5)		
macro	52 (5.5)	55 (3.8)		
peripheral	48 (5.0)	41 (2.9)		
punctate echogenic foci	521 (54.9)	282 (19.8)		

We further divided the positive group into two subgroups, PTC1 and PTC2. In comparing these two subgroups, CSAR ≥ 1 showed a significant difference to predict CLNM in the PTC2 subgroup (**Table 3**).

**Table 3:** Univariate analysis of PTC1 and PTC2 in the positive group.

Variable	Cross-sectionalaspect ratio≥1	longitudinal section aspect ratio≥1	Statistics	P
			x^2^ = 14.455	P<0.001
PTC1+CLNM (445)	281	205		
PTC2+CLNM (504)	181	70		

Clinical information as well as ultrasonographic measurements and characteristics were included in the binary linear regression analysis. Aspect ratio was included in cross-sectional and longitudinal section aspect ratio. The results showed that gender, age, maximum tumor diameter and volume, CSAR, location, echogenicity, margin, and echogenic foci were independent risk factors for CLNM (**Table 4**).

**Table 4:** Logistic regression analysis of correlative factors with CLNM in PTC.

Variable	OR	95% CIs	P
Overall (N=949, n=1427)			
Gender	1.69	1.45~1.97	P < 0.001
Age	0.97	0.96~0.97	P < 0.001
Maximum tumor diameter	1.07	1.05~1.09	P < 0.001
Tumor volume	0.94	0.91~0.98	P = 0.002
Cross-sectional aspect ratio	1.52	1.08~2.12	P = 0.016
Longitudinal section aspect ratio	1.04	0.74~1.46	P = 0.836
Location	1.07	1.03~1.11	P=0.001
Echogenicity	2.68	2.14~3.36	P < 0.001
Margin	1.68	1.56~1.81	P < 0.001
Echogenic foci	1.56	1.48~1.64	P < 0.001

N, The number of patients for the positive group; n, The number of patients for the negative group.

**Figure 2:**
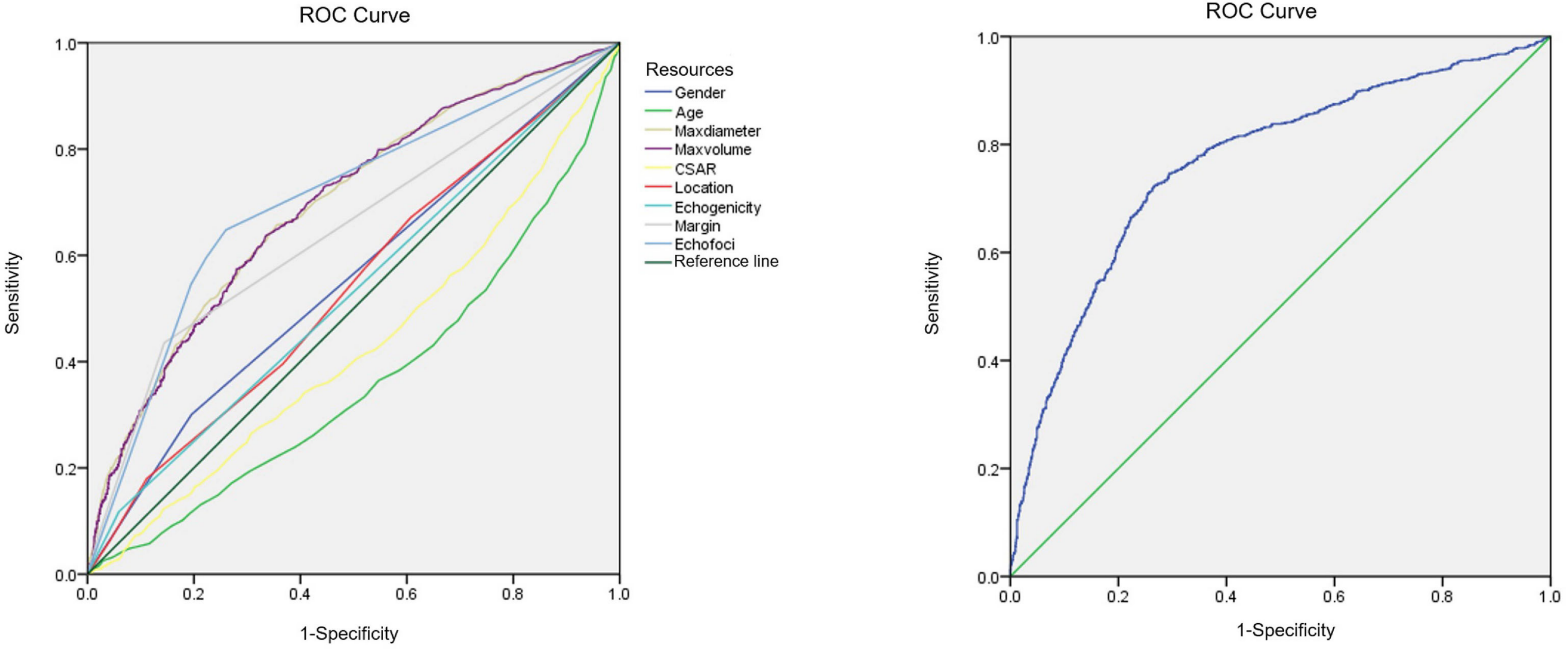
ROC curves for independent correlative factors and equation with CLNM in PTC.

Binary linear regression analysis and the ROC curves were used to obtain the independent correlative factors for CLNM. The AUC, specificity, and sensitivity of maximum tumor diameter were 0.70, 64.5%, and 65.7%, respectively. For tumor volume, the AUC, specificity, and sensitivity were 0.69, 66.4%, and 63.8%, respectively. For margin, the AUC, specificity, and sensitivity were 0.65, 85.6%, and 43.5%, respectively. For echogenic foci, the AUC, specificity, and sensitivity were 0.70, 74.0%, and 64.8%, respectively. To consider CLNM in PTC patients as a dependent variable and independent risk factors as covariates, a regression equation of CLNM was deduced from the parameters: Y = −3.657+0.063*X1-0.061*X2+0.519*X3+0.444*X4. In this equation, Y = CLNM, X1 = maximum tumor diameter, X2 = tumor volume, X3 = margin, and X4 = echogenic foci. The ROC curve was used to evaluate the regression model constructed by combining four independent correlative factors (maximum tumor diameter and volume, margin, and echogenic foci), in which the value of AUC, specificity, and sensitivity was 0.77, 73.4%, and 72.3%, respectively (**Table 5**, **Figure 2**). The AUC differences among the prediction model and the dependent variables have statistical significance (P < 0.001).

**Table 5:** ROC analysis of the independent variables in identifying CLNM in PTC.

Variable	AUC	95% CIs	Specificity	Sensitivity
Overall (N=949, n=1427)				
Gender	0.55	0.54~0.57	80.5%	30.1%
Age	0.37	0.35~0.38	99.9%	0.2%
Tumor maximum diameter	0.70	0.68~0.71	64.5%	65.7%
Tumor volume	0.69	0.68~0.71	66.4%	63.8%
Cross-sectional aspect ratio	0.43	0.41~0.45	99.6%	0.2%
Location	0.54	0.52~0.55	88.8%	18.0%
Echogenicity	0.53	0.51~0.55	94.1%	11.8%
Margin	0.65	0.63~0.66	85.6%	43.5%
Echogenic foci	0.70	0.69~0.72	74.0%	64.8%
Equation	0.77	0.75~0.78	73.4%	72.3%

N, The number of patients for the positive group; n, The number of patients for the negative group.

According to the differences in clinical and ultrasonic characteristics using Kruskal-Wallis analysis, maximum tumor diameter and volume, cross-sectional and longitudinal aspect ratio, margin, and echogenic foci were statistically significant (P. < 0.05). The comparisons between the positive subgroups are shown in **Table 6**.

**Table 6:** Kruskal-Wallis analysis of the positive subgroups with CLNM in PTC.

Variable	Central (n=669)	Lateral (n=70)	Both (n=210)	Statistics	P
Gender (male/female)	197/472	17/53	76/134	x^2^ = 4.821	P = 0.090
Age	41.1 ± 11.5	44.2 ± 13.5	40.1 ± 13.0	x^2^ = 5.654	P = 0.059
Tumor maximum diameter	11.2 ± 7.3	15.9 ± 9.5	19.0 ± 11.4	x^2^ = 130.278	P < 0.001
Tumor volume	0.9 ± 2.2	2.8 ± 6.2	3.2 ± 6.2	x^2^ = 132.410	P < 0.001
Cross-sectional aspect ratio	1.0 ± 0.3	1.0 ± 0.3	0.9 ± 0.2	x^2^ = 16.378	P < 0.001
Longitudinal aspect ratio	0.9 ± 0.3	0.9 ± 0.2	0.8 ± 0.3	x^2^ = 41.625	P < 0.001
**Echogenicity**				x^2^ = 0.474	P = 0.789
Hyperechoic or isoechoic	10 (1.5)	0 (0)	6 (2.9)		
Hypoechoic	603 (90.1)	66 (94.3)	187 (89.0)		
Very hypoechoic	56 (8.4)	4 (5.7)	17 (8.1)		
**Margin**				x^2^ = 69.606	P < 0.001
smooth or ill-defined	436 (65.2)	23 (32.9)	74 (35.2)		
lobulated or irregular	199 (29.7)	43 (61.4)	117 (55.7)		
extra-thyroid extension	34 (5.1)	4 (5.7)	19 (9.1)		
**Echogenic foci**				x^2^ = 35.508	P < 0.001
none or large comet-tail artifacts	274 (41.0)	17 (24.3)	37 (17.6)		
macro	36 (5.4)	1 (1.4)	15 (7.1)		
peripheral	27 (4.0)	7 (10.0)	14 (6.7)		
punctate echogenic foci	332 (49.6)	45 (64.3)	144 (68.6)		

## Discussion

With the application of high frequency ultrasound, the incidence of thyroid lesions in adults can be up to 60%–70% (10). PTC is the most common malignant thyroid tumor, and lymph node metastasis of PTC is associated with the diameter, location, number, and invasive growth of the primary tumor (9, 11). The sensitivity of ultrasound for the evaluation of central and lateral compartment lymph nodes is around 60% (9) In this study, compared with cN1 and pN1, the detection rate of cervical lymph node metastases was 62.2% (590/949). CLNM in PTC generally first occurs in the central region and then progresses to the lateral region (12), which is most common in area VI, followed by areas III and IV (13). However, not all PTC lymph node metastases follow this pattern and instead skip, where lymph node metastasis occurs in the lateral neck region and no metastasis is identified in the ipsilateral central region (14). Some studies have reported that the rate of lymph node skip metastasis is 3.0%–21.8% (15). Only unifocal PTC was included in this study and the lymph node skip metastasis rate was 7.4% (70/949).

Heaton et al. (16) reported that women and elderly patients were at higher risk of PTC, while men and younger patients were at higher risk of CLNM. In this study, CLNM occurred in 51.0% (290/569) of male patients, and occurred in only 36.5% (659/1807) of female patients, which also suggests that male patients have a higher risk of lymph node metastasis. We used 55 years as the threshold in this study according to the TNM staging system for thyroid cancer (17), and 42.4% (827/1952) of patients who were younger than 55 years had CLNM. Among those who were 55 years or older, lymph node metastases occurred in only 28.8% (122/424) of patients in this study.

Maximum tumor diameter is an important reference index for PTC treatment protocols and for range of surgery (18). The results of this study demonstrated that the average maximum tumor diameter in the positive group was about 1.6 times that in the negative group (13.3 mm vs. 8.5 mm, respectively). The tumors appeared to be ellipsoid, and therefore volume as the evaluation parameter made the result more objective and scientific. The average tumor volume in the positive group was about 3.2 times that in the negative group (1.6 ml vs. 0.5 ml, respectively). For larger tumors, cervical lymph nodes should be examined in order to improve the detection rate of CLNM. In particular, in patients with large tumors, the presence of central and lateral lymph node metastasis should be determined in advance.

TI-RADS was mainly adopted for the differential diagnosis of benign and malignant thyroid neoplasms. The consistent conclusion of the association between lymph node metastasis and gender, age, and sonographic characteristics was difficult to achieve (19–21). This study only included unifocal PTC and was conducted as a stratified study to comprehensively evaluate the risks of CLNM in PTC. This was combined with clinical information, ultrasonographic measurements, and TI-RADS to obtain a more complete and effective result.

PTC can occur in any part of the thyroid, including bilateral lobes and the isthmus. For the isthmus, the incidence of thyroid cancer is 2.5%–9.2% (22). Studies have found that PTC of the isthmus is more likely to invade the thyroid capsule and surrounding tissues, compared to lateral lobe PTC (18, 23). This is mainly because the area of the isthmus tumor in contact with thyroid capsule is relatively large, and can therefore easily invade the capsule or break through the capsule to invade surrounding tissues, leading to CLNM. Previous studies (24) have suggested that tumors located in the middle or lower pole of thyroid have increased risk of CLNM. In this study, the incidence of PTC lymph node metastasis was higher in the middle pole than in the upper pole, lower pole, or isthmus (middle, 42%; upper, 30.7%; lower, 21.1%; isthmus, 6.2%).

An aspect ratio ≥ 1 is a highly specific index for the diagnosis of malignant thyroid nodules (25, 26). According to previous literature, the results of association between aspect ratio and CLNM in PTC were not consistent. Some studies (27, 28) have suggested that CLNM was prone to occur with an aspect ratio > 1 and that CLNM was the risk factor. One study (29) reported that no statistical significance could be seen in the prediction of lateral CLNM with an aspect ratio ≥ 1. According to the morphology of a thyroid lesion, we divided the aspect ratio into cross-sectional and longitudinal section aspect ratios. Aspect ratio had statistical significance in univariate analysis, and while we used the binary logistical analysis, there was no significant difference for longitudinal section aspect ratio. The main reason was that PTMC patients comprised 63.9% (1518/2376) of the study participants, and just 29.3% (445/1518) were in the positive group. We compared CSAR and longitudinal section aspect ratio for PTC1 and PTC2 groups, and it could be concluded that the CSAR had a better predictive value for CLNM in PTC2. There was less relevant literature with the association between cross-sectional and longitudinal section aspect ratio in predicting CLNM, especially for PTC2. Additional studies are needed in the future for clarification.

When malignant tumors grow rapidly, the cancer cells continue to invade outward and the incidence of lymph node metastasis is increased (16, 30). Margin is traditionally used to analyze the invasiveness of a tumor. Nodules with high invasiveness show irregular and lobulated boundaries, while smooth boundaries generally indicate low invasiveness and slow growth (31). In this study, univariate analysis and logistic regression analysis both showed a good association between margin and CLNM in PTC. This is consistent with previous literature reports (20, 24). Lesions with lobulated or irregular shape occurred more often in the lateral metastasis and both metastasis groups than the central metastasis group by Kruskal-Wallis analysis.

Echogenic foci are classified as micro-calcification, macro-calcification, or ring calcification around the nodules on the basis of 1 mm (32). Micro-calcification can reflect the psammoma bodies in pathology, which results from calcification and necrosis of cancer cells and is a specific indicator for the diagnosis of PTC (33); it is also significantly related to lymph node metastasis. Continuous follow-up studies found that CLNM was more likely to occur in PTC with micro-calcification (31, 34). In this study, 54.9% (521/949) of lesions with CLNM had punctate echogenic foci. Among the negative group, punctate echogenic foci occurred in only 19.8% (282/1427) of lesions. For the positive subgroups, echogenic foci also showed a significant difference (P < 0.001). Therefore, ultrasonography can better predict the risk of CLNM in PTC using the different types of calcifications.

TI-RADS comprehensively evaluated tumors according to sonographic characteristics of thyroid nodules. Its scoring and grading system were used for the differential diagnosis of benign and malignant nodules, and it would be of great value for further determination of diagnosis and treatment protocols. In this study, ultrasonographic measurements and characteristics were included for logistic regression analysis to establish a prediction model for CLNM with the AUC of 0.77, a specificity of 73.4% and a sensitivity of 72.3%. Sun et al. (35) reported that they used gender, age, max tumor diameter, number of nodules, and cervical lymph node detected by ultrasound as covariates; CLNM was a dependent variable, and a prediction model was acquired with a specificity of 80.8% and a sensitivity of 59.8%. Zou et al. (36) reported that the area under the ROC curve was 0.758 for the preoperative prediction of lymph nodes posterior to the right recurrent laryngeal nerve metastasis, and used five independent correlative factors (age, male, tumor diameter, US-detected lateral compartment lymph node metastasis, and microcalcifications) for evaluation. Liang et al. (37) found that central lymph node metastasis was associated with male gender, younger age (< 45 years), extrathyroidal invasion, multifocality, and lateral lymph node metastasis in PTMC. In our study, central neck metastasis, lateral neck metastasis, and both central and lateral neck metastasis were analyzed and compared for PTC, including PTMC. We also divided the aspect ratio into cross-sectional and longitudinal section aspect ratios, and CSAR had a more effective predictive value for larger thyroid tumors. The result has reference value and also demonstrates the importance of comprehensive evaluation of ultrasound in clinical practice.

This study has some limitations. First, this was a retrospective study including unifocal PTC and lymph node dissection performed in the central area, which may cause selection bias. Second, cases with metastases in lateral locations were not adequate, and large samples are required to study cervical metastases in different locations. For skip lateral lymph node metastases, more effective preoperative assessment should be adopted. Finally, this is our preliminary study for a larger future study of PTC patients. In future research, we will add detailed clinical and pathological staging, subdivided pathological types, and machine learning models.

## Conclusions

In conclusion, based on PTC pathology, the correlations between gender and age, as well as ultrasonographic measurements and characteristics were analyzed in order to assess a good clinical value for preoperative evaluation of the risk of lymph node metastasis. For PTC patients with high-risk factors such as age < 55 years, male, larger tumor and punctate echogenic foci, preoperative lymph node examination should be conducted in detail. This examination should include a cervical lymph node sonographic scan by an experienced sonographer (with experience of more than 10 years) and CT scan of the neck, which will cover a wide range of thyroid cancer population and help develop the most reasonable clinical treatment protocol.

## Data availability statement

The original contributions presented in the study are included in the article/**Supplementary Material**. Further inquiries can be directed to the corresponding authors.

## Ethics statement

The studies involving human participants were reviewed and approved by the Ethics Committee of Cancer Hospital of the University of Chinese Academy of Sciences (Zhejiang Cancer Hospital), Zhejiang Xiaoshan Hospital and Hangzhou Cancer Hospital. The patients/participants provided their written informed consent to participate in this study.

## Author contributions

DX had full access to all data and took responsibility for the integrity and accuracy of the data analysis. LZ and JY were major contributors in writing the manuscript. DO participated in collection and management of the data. LW, ML and ZL contributed to analyze and interpret the data. All authors contributed to the article and approved the submitted version.

## Funding

This study was supported by National Natural Science Foundation of China (NO. 82071946) and Zhejiang Provincial Natural Science Foundation of China (NO. LSD19H180001). DX is responsible for these two funds.

## Acknowledgments

The authors thank Chen Cui, PhD for reviewing the manuscript and providing support with data analysis. This manuscript has been released as a pre-print at Research Square, DOI:10.21203/rs.3.rs-839643/v1.

## Conflict of interest

The authors declare that the research was conducted in the absence of any commercial or financial relationships that could be construed as a potential conflict of interest.

## Publisher's note

All claims expressed in this article are solely those of the authors and do not necessarily represent those of their affiliated organizations, or those of the publisher, the editors and the reviewers. Any product that may be evaluated in this article, or claim that may be made by its manufacturer, is not guaranteed or endorsed by the publisher.

## Abbreviations

PTC, Papillary thyroid carcinoma; CLNM, Cervical lymph node metastasis; PTMC, Papillary thyroid microcarcinoma; ROC, Receiver operating characteristic; CSAR, Cross-sectional aspect ratio; AUC, Area under the curve; TI-RADS, Thyroid imaging report and data system; CT, Computed tomography; SD, Standard deviation; OR, Odds ratio; CI, Confidence interval.
